# Dexterous Manipulation for Multi-Fingered Robotic Hands With Reinforcement Learning: A Review

**DOI:** 10.3389/fnbot.2022.861825

**Published:** 2022-04-25

**Authors:** Chunmiao Yu, Peng Wang

**Affiliations:** ^1^Institute of Automation, Chinese Academy of Sciences, Beijing, China; ^2^School of Artificial Intelligence, University of Chinese Academy of Sciences, Beijing, China; ^3^CAS Center for Excellence in Brain Science and Intelligence Technology, Chinese Academy of Sciences, Shanghai, China; ^4^Centre for Artificial Intelligence and Robotics, Hong Kong Institute of Science and Innovation, Chinese Academy of Sciences, Hong Kong, China

**Keywords:** dexterous manipulation, multi-fingered robotic hand, reinforcement learning, learn from demonstration, sim2real

## Abstract

With the increasing demand for the dexterity of robotic operation, dexterous manipulation of multi-fingered robotic hands with reinforcement learning is an interesting subject in the field of robotics research. Our purpose is to present a comprehensive review of the techniques for dexterous manipulation with multi-fingered robotic hands, such as the model-based approach without learning in early years, and the latest research and methodologies focused on the method based on reinforcement learning and its variations. This work attempts to summarize the evolution and the state of the art in this field and provide a summary of the current challenges and future directions in a way that allows future researchers to understand this field.

## Introduction

Robotics has been a topic of interest for researchers for decades, and dexterous manipulation is one of the hottest these days. Although some simple tasks in the industrial environment have been solved, we also wish the robot can help us in some unstructured environments such as the domestic environment (e.g., helping blind people with daily routines) and some dangerous environments (e.g., nuclear decommissioning). Hence, the ability to operate with the dexterity of the robot is necessary. There are several definitions of dexterous manipulation problem, among which the one proposed by Bicchi (2000) is thorough and widely accepted: dexterous manipulation is the capability of changing the position and orientation of the manipulated object from a given reference configuration to a different one, arbitrarily chosen within the hand workspace.

**Figure 1:**
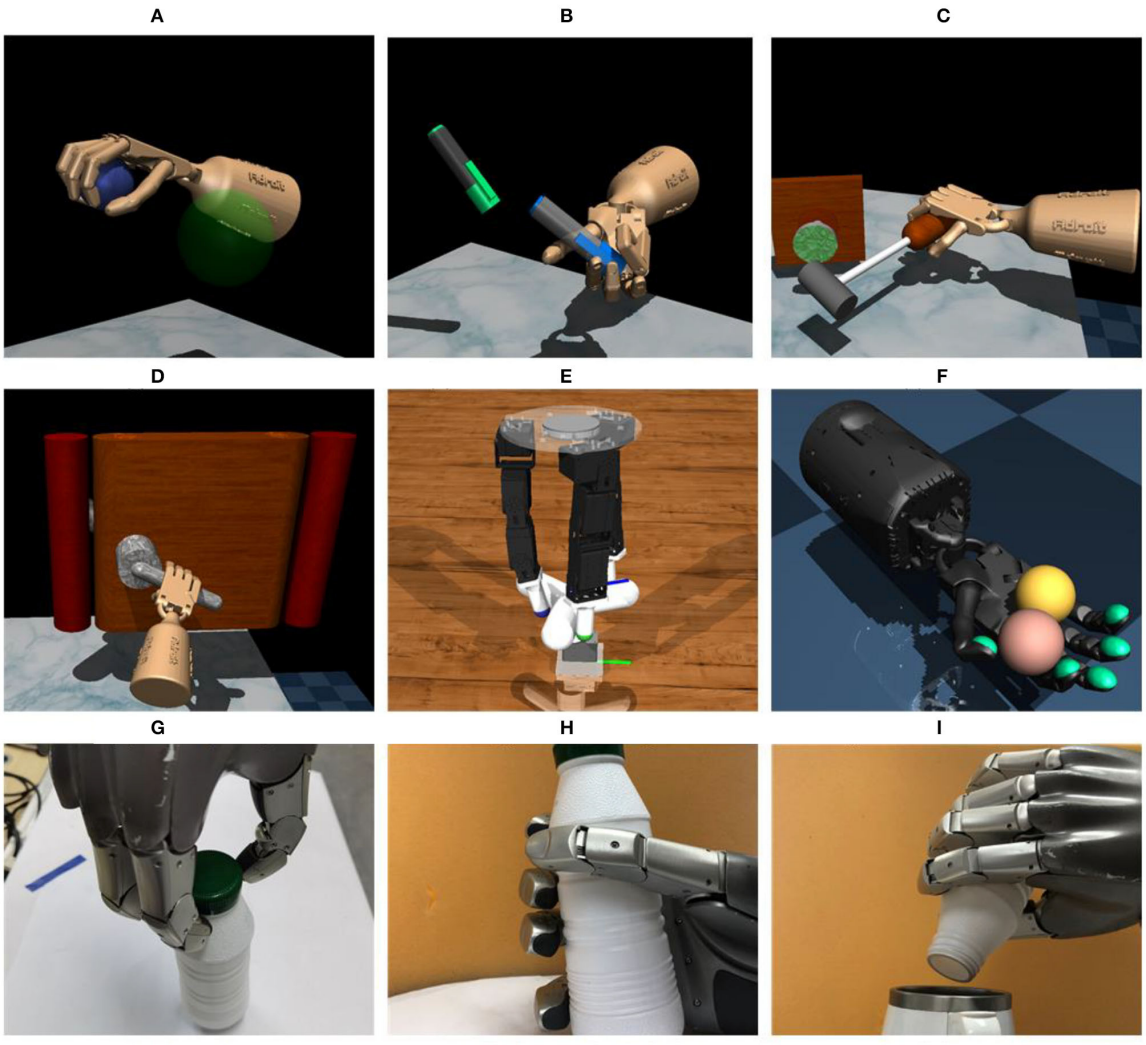
Typical tasks of dexterous manipulation with a multi-fingered hand. **(A)** Relocation, **(B)** Reorientation & Relocation, **(C)** Tool use, **(D)** Door opening, **(E)** Valve turning, **(F)** In-hand manipulation, **(G)** Screwing, **(H)** Dexterous manipulation, and **(I)** Pouring.

In a structured environment where the shape of the objects is unaltered, the simple gripper is sufficient for simple tasks such as the pick-and-place task, and the gripper has more advantages in these tasks on account of its low price, easy control, and strong robustness. However, the dexterity of parallel claws is limited and they are not adapted to various objects and tasks. One solution is designing specific end-effectors for different objects and tasks. In a structured environment, this method is effective, but when facing a complex unstructured environment where one robot needs to deal with a lot of tasks and one robot needs to carry different end-effectors for different tasks, it is unpractical. Also, someone people argued that a dexterous arm with a simple gripper may be sufficient (Ma and Dollar, 2011). They pointed out that in some cases where the hand is for simple grasping and the arm is for manipulation, a dexterous arm with a simple gripper is sufficient and appropriate for many manipulation tasks. However, for some complex tasks such as in-hand manipulation, a simple gripper is not sufficient and a multi-fingered dexterous hand is, therefore, necessary. Figure 1 shows the typical tasks of dexterous manipulation with multi-fingered robotic hands including pouring (Qin et al., 2021), dexterous grasping (Li et al., 2014), object relocation (Rajeswaran et al., 2018), and so on, which are difficult or impossible to be accomplished by simple manipulators.

A dexterous hand can greatly improve dexterity and increase the workspace of the system. Additionally, the application of the dexterous hand can reduce the energy required for the task due to the lower feedback gains required as opposed to a full arm.

When mentioning a dexterous manipulator, the first thing that comes to mind is the human hand. Even some philosophers deem that it is the dexterity of the human hand that leads to human intelligence. Therefore, it is no surprise that most robot hands designed for dexterous manipulation are similar to the human hand in both shape and structure. The past several decades have seen the emergence of many dexterous multi-fingered hands. In 1984, the Center for Engineering Design at the University of Utah, and the Artificial Intelligence Laboratory at the Massachusetts Institute of Technology designed the UTAH/MIT hand with three fingers and a thumb aiming at machine dexterity (Jacobsen et al., 1986), and later HIT developed the DLR/HIT Hand II (Liu et al., 2008). Also, there are some commercial products such as the Shadow Hand (2005) and SimLab (Allegro hand). Apart from the dexterous humanoid robotic hands, some simpler robotic manipulators with fewer fingers and a lower dimension of freedom (DoF) are also designed for better robustness and lower price (Zhu H. et al., 2018; Wüthrich et al., 2021). Some common multi-fingered robotic hands and some important parameters are shown in Table 1. However, up to now, the dexterity of the human hand is still unparalleled and it is scarcely possible to emulate the level of its functionality.

**Table 1:** Typical dexterous hands.

**Name**	**TriFinger (Wüthrich et al., 2021)**	**Dclaw (Zhu H. et al., 2018)**	**Utah/MIT (Jacobsen et al., 1986)**	**Allegro (Allegro hand)**	**Shadow (Shadow Hand, 2005)**	**DLR/HIT II (Liu et al., 2008)**
Picture	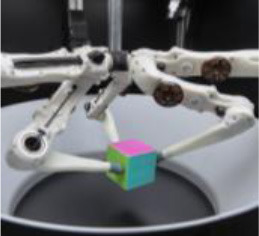	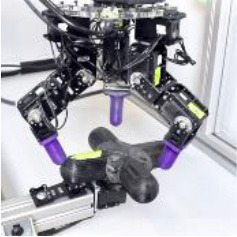	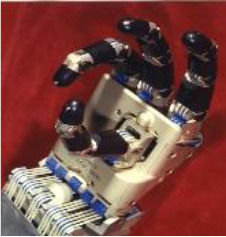	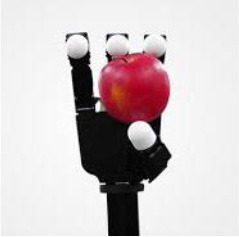	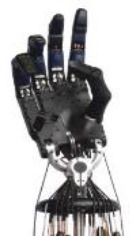	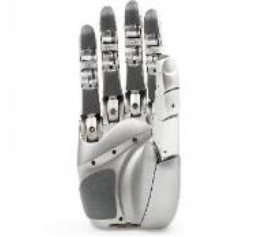
Fingers	3	3	4	4	5	5
DoF	9	9	16	16	24	15

The current applications of robotic hands in the factories still use traditional engineering and analysis techniques. Typically, some robots with simple end-effectors are widely used in the manufacturing industry for packaging and palletizing. Similarly, agricultural robotic hands with several end-effectors and painting robotics are good examples for the application of robotic hands in the structured industry environment. Although the dexterous manipulation problem has been studied extensively, the application of the learning-base methods in this review still remain at the laboratory level, which is not sufficient for unstructured environment such as businesses and homes.

Although the mechanical design of smart manipulators has improved greatly, the actual dexterity of the robotic hands is far inferior to that of the human hand. On the one hand, lots of sensors and actuators of the human hand makes it almost impossible to design a robotic hand which is similar to the human hand (Billard, 2019), and on the other hand, the control of the robotic hand to realize dexterous manipulation is still an urgent problem to solve. Before 2000, the approach was based on the kinematics and dynamics of manipulating an object with the fingertips dominating the area. This approach requires the complete information of the manipulator kinematics, dynamics, interaction forces, high-fidelity tactile, and/or joint position sensors available on-board the robot. However, the accurate model of the environment and the object is not or partly available in the real world. Moreover, even though the information is available, the algorithm must change as the object or the manipulator changes. Hence, in the real world, the model-based approach has certain limitations.

Recently, the power of artificial intelligence has attracted the attention of many researchers. Deep learning has even reached a level that exceeds that of humans in certain fields, such as computer vision, so the robot can extract generalized features autonomously (LeCun et al., 2015; Duan et al., 2021; Wei et al., 2021, 2022; Li et al., 2022). Deep learning is better at classification and prediction problems and so on. But the application of deep learning is still short of the entire system model. In contrast, reinforcement learning (RL) is more suitable for dealing with the sequential decision problem. Therefore, the combination of deep learning and reinforcement learning called deep reinforcement learning is proposed to realize more complicated problems involving perception and decision making. Dexterous manipulation is a typical decision-making problem, so deep reinforcement learning, as it were, dominated the area in recent years. However, the application of deep reinforcement learning to dexterous manipulation has some disadvantages. First, the sparse reward makes the training hard, and for complex tasks, it is time-consuming and the requirement of computing power is high. Furthermore, deep reinforcement learning requires many samples obtained by trial and error, which are nearly unavailable in a robotic system. To solve this problem, besides the improvement of the RL algorithm, usually two solutions are considered: learning from demonstration and transferring the policy learned in simulation to reality. These two approaches will greatly enhance the efficiency of the algorithm.

There are already several works reviewing the robot manipulation domain (Billard, 2019; Cui and Trinkle, 2021), reinforcement learning for the robot (Hua et al., 2021; Zhang and Mo, 2021), and dexterous manipulation only (Prattichizzo et al., 2020). However, as far as we know, a survey focusing on dexterous manipulation with multi-fingered robotic hands with reinforcement learning has never been presented before. Here, we present a review of this domain including the method based on dynamic analysis in the earlier years and the reinforcement learning-based method in recent years. Although the method based on reinforcement learning is the core of this paper, we think the method based on dynamic analysis is necessary for readers to understand the dexterous manipulation problem.

**Figure 2:**
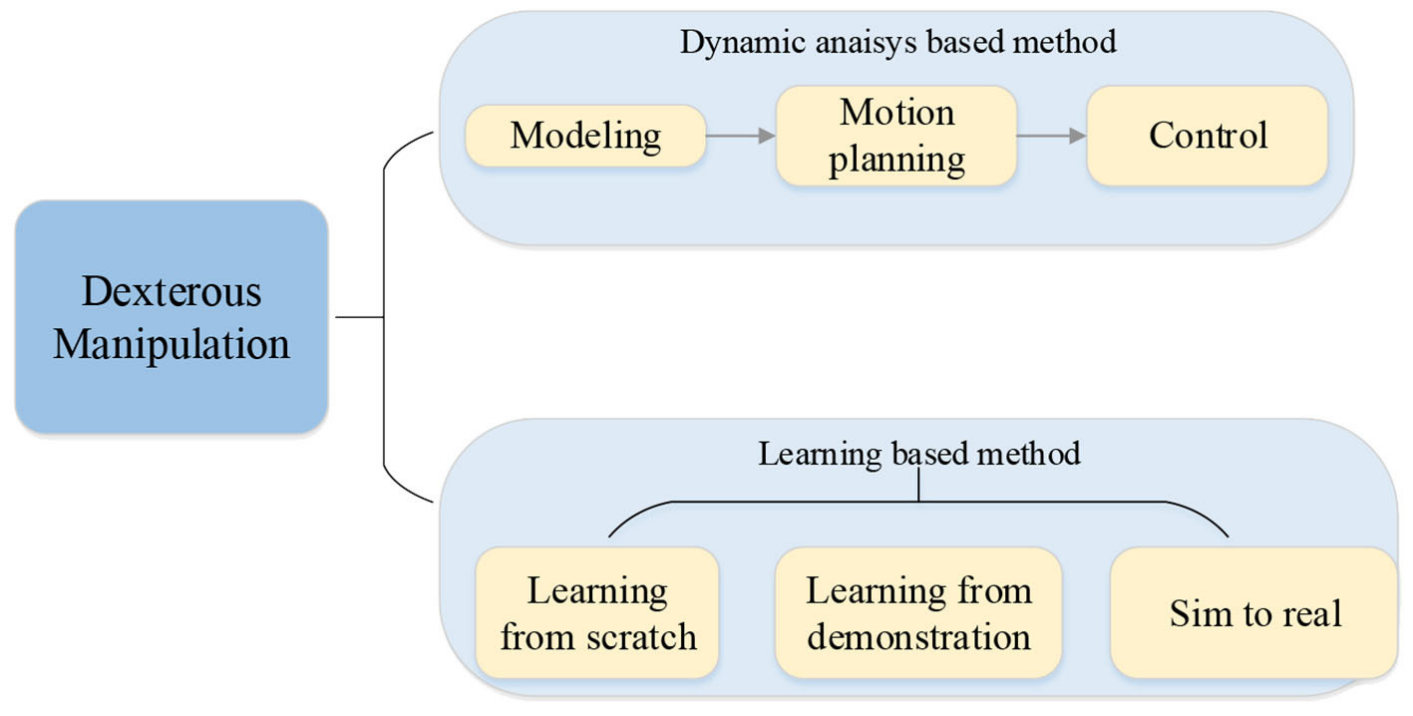
Overall presentation of this work.

The main contribution of this paper is presenting a state-of-the-art review focused on the dexterous manipulation problem of multi-fingered robotic hands with reinforcement learning. The paper first reviews the model-based approach without learning including the basic modeling, planning, and control. Further, the methods based on deep reinforcement learning, reinforcement learning from demonstration, and transfer learning from simulation to reality are summarized and analyzed thoroughly. Finally, challenges and future research directions are proposed. The main topics discussed in this article are shown in Figure 2.

The rest of this article is organized as follows. After this introductory section, in Section Dexterous Manipulation for Multi-Fingered Robotic Hand Based on Modeling, Planning, and Control, we introduce the basic theory of dexterous manipulation including the model of the multi-fingered robotic hands and the object and the model-based approach for dexterous manipulation. Section Dexterous Manipulation for Multi-Fingered Robotic Hands With Reinforcement Learning focuses on the dexterous manipulation with reinforcement learning, including the application of reinforcement learning, the combination of reinforcement learning, and learning from demonstration and deploying the learned policy in simulation to the real world. At the same time, we also discuss the characteristics of the approaches mentioned in this paper. Section Challenges and Future Research Directions describes the current limiting factors in manipulation and look forward to the further development of dexterous manipulation.

## Dexterous Manipulation for Multi-Fingered Robotic Hand Based On Modeling, Planning, and Control

**Figure 3:**
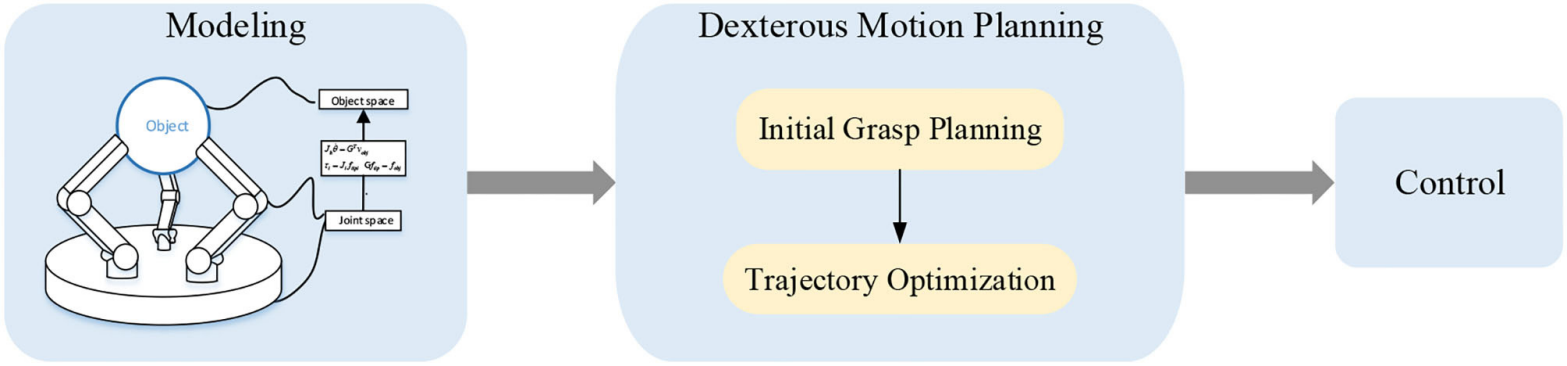
Method based on an accurate model of multi-fingered hand and object.

The dexterous problem can be described as determining the contact points and the forces/torques that should be exerted upon the object and planning a trajectory to control the end-effector to accomplish a specific task. In this section, we will introduce the basic theories of dexterous manipulation including the models of contacts, positions, forces, and velocities; motion planning and the control framework for dexterous manipulation. The progress of the model-based approach including modeling, dexterous motion planning, and control are depicted in Figure 3.

### Modeling of Multi-Fingered Robotic Hands and Objects

Usually, an object-centered point of view is adopted for describing the dexterous manipulation problem. The formulations are in terms of the object to be manipulated, how it should behave, and what forces should be exerted upon it. Therefore, the relationship of the desired forces/torques on the object and the required contact forces and the relationship of the required contact forces and the joint torques is required. Typically, the model of contact between the object and the fingertip can be seen as point-contact and the model of the robotic hand can be seen as a set of kinematic chains consisting of links connected by joints. The most popular method for formulating the forward kinematics of robots is the D-H method. Specifically, four D-H parameters are used for the transformation between two co-ordinate systems. More details about the D-H convention and the point-contact model can be seen in (Spong et al., 2006) and (Okamura et al., 2000), respectively.

In addition to maintaining the contacts during the manipulation process, rolling and sliding may sometimes occur during manipulation. Although sliding in some tasks is not allowed, the sliding mode is necessary when exploring an unknown object or changing the pose of a grasp to maintain control of the object. More details about rolling and sliding can be seen in Montana (1988) and Kao and Cutkosky (1992), respectively. However, sliding is rarely considered in the early years due to lack of reliable tactile sensors to keep track of the contact locations on the fingertips and indicate the onset of slip.

### Dexterous Motion Planning of Multi-Fingered Robotic Hand

Typically, the dexterous manipulation problem can be divided into two parts, namely initial grasp planning and trajectory optimization which will be discussed, respectively, in this subsection.

#### Grasp Planning of Multi-Fingered Robotic Hands

To deal with dexterous manipulation, the first thing to be considered is stable grasping. Grasping generally consists of two phases: a planning phase and a holding phase. In the planning phase, the finger contact point locations are decided and the object is grasped stably in the holding space. Two important problems are considered for the two phases accordingly: the selection of feasible locations of contact and optimal contact forces.

#### Selection of Feasible Locations of Contact

Two important concepts describing the stability of a given grasp are force-closure and form-closure. We refer the readers to Bicchi (1995) for more details about force-closure and form-closure. However, force-closure is only the bottom-most condition to satisfy and not enough for a stable and desired grasp. Furthermore, in a specific task, there would be many configurations that achieve force-closure, so the problem that which one should be adopted is very important. Being on the safer side, an intuitive measurement is to apply less force on the object, resulting in a better grasp effect. The first one who proposed this idea was Kirkpatrick et al. (1992), and Ferrari and Canny (1992) improved it later. Similarly, for different consideration factors, a few metrics were proposed, such as task-oriented metrics (Hsu et al., 1988), eigenvalue decomposition-based metric (Bruyninckx et al., 1998), and metrics considering different issues (Lin and Burdick, 1999; Lin et al., 2000; Roa and Suárez, 2009). However, getting optimal contact locations through appropriate metrics and optimization methods is difficult due to that the quality measure is typically a non-convex (and non-linear) function. Besides the optimization approach, some researchers used a knowledge-based approach (Cutkosky, 1989; Stansfield, 1991) to get a suitable grasp.

#### Selection of Optimal Contact Forces

To generate a great grasp, we should plan not only the locations of the contact points but also the force exerted to the object on the contact. In early works, the friction constraint was linearized and the coefficient of friction was estimated conservatively to avoid instability and considering the problem as a non-linear programming problem (Nakamura et al., 1989; Nahon and Angeles, 1991; Al-Gallaf and Warwick, 1995). However, such methods are offline, and considering the problem in a non-linear context was also proposed for online implementation (Buss et al., 1996). These computed forces are then used in the low-level force servo mechanism to produce a desired force behavior in the object.

#### Trajectory Optimization for Dexterous Manipulation With a Multi-Fingered Robotic Hand

For relatively simple tasks, the contact points remain the same during the manipulation, so after getting the desired grasp configuration and contact forces, the task can be achieved by controlling the robot arm. However, for more complex tasks such as in-hand manipulation, one grasp is not sufficient. Therefore, a trajectory of grasps which links the initial grasp and the desired grasp is required.

The methods proposed in the dexterous manipulation problem are typically derived from the legged locomotion problem. However, the methods used in the legged locomotion are not suitable for hand movement control due to the high dimensions of the search space. A representative work proposed by Mordatch et al. (2012a) is an extension of contact-invariant optimization (CIO) (Mordatch et al., 2012b) which was used for character animation originally. However, the CIO is an offline method and time-consuming. In practice, online planning (or Model-Predictive Control) is more desirable (Kumar et al., 2014), where a trajectory of the control signal is optimized and the joint space trajectories are obtained through inverse kinematic (IK). For solving (Sundaralingam and Hermans, 2017) the in-grasp manipulation problem more directly, get a joint space trajectory without the process of IK. However, this approach requires maintaining the contacts, which is only a part of the whole dexterous manipulation process. With this in mind, Sundaralingam and Hermans (2018) presented a planner for reorientation of the object through finger gaiting and in-grasp manipulation alternately. Similarly, Chen C. et al. (2021a) proposed TrajectoTree, a method based on contact-implicit trajectory optimization (CITO). Unlike the optimization method, the concept of motion primitives is also accepted widely (Chen C. et al., 2021b; Yoneda et al., 2021). The phase of motion planning is the core of dexterous manipulation. However, only under certain assumptions can these approaches work, such as assuming that the shape and mass of the object are known and the contacts remain during the manipulation process (Sundaralingam and Hermans, 2017). Also, some approaches can only be applied to planar objects (Chen C. et al., 2021a). At the same time, most of these methods are only tested in simulation. From what has been discussed above, the approaches based on trajectory optimization have many limitations for achieving dexterous manipulation with multi-fingered robotic hands in the real world.

### The Control of Multi-Fingered Robotic Hand for Dexterous Manipulation

The control of multi-fingered robotic hands for dexterous manipulation can typically be divided into three levels. The high-level control includes grasp planning and motion planning which have been discussed thoroughly. The middle-level control, which is relatively unpopular compared to the other two levels, includes event detections and phases transitions. Hence, only a few researchers focus on this problem (Johansson and Westling, 1991; Eberman and Salisbury, 1994; Hyde et al., 1997; Hyde and Cutkosky, 1998).

The low-level control is a primary part of the dexterous manipulation problem and has received a lot of attention. Trajectory tracking in free space and precise force control in constrained space should be both taken into consideration. During tracking in free space, position control is enough because the robot hand does not make contact with the object at this stage. During the contact stage, position control and force control are both important for precise force. Taking both position control and force control into account, several control algorithms were put forward such as simple hybrid position/force control which is widely used (Raibert and Craig, 1981; Xiao et al., 2000), impedance control (Hogan, 1984), and the combination of hybrid position/force control and impedance control (Anderson and Spong, 1988). The impedance control can solve the problem of discontinuity by the change of the control mode, so it has attracted much attention of researchers (Goldenberg, 1988; Kelly and Carelli, 1988; Kelly et al., 1989). The combination can furthermore be considered as the distinction between force-controlled subspaces and position-controlled subspaces.

## Dexterous Manipulation for Multi-Fingered Robotic Hands With Reinforcement Learning

**Figure 4:**
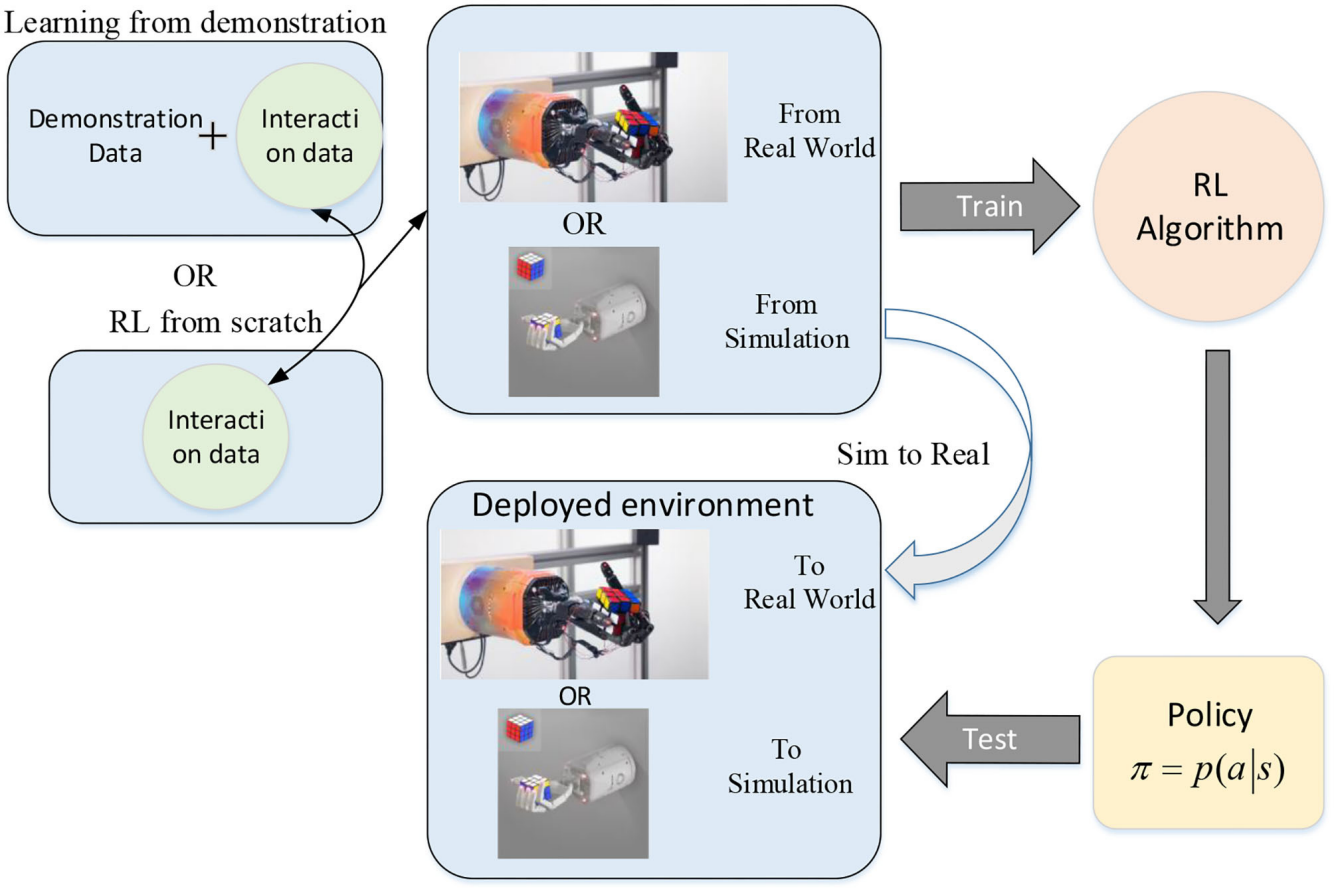
Dexterous manipulation with a multi-fingered hand through reinforcement learning (part of this picture comes from [89]).

Given that the complete model of the objects and robotic hand is difficult to obtain in an unstructured environment and programming robots require a high degree of expertise, the methods mentioned above are not sufficient for a more complicated environment and tasks. The development of machine learning, especially reinforcement learning, provides new solutions to the problem of dexterous manipulation with multi-fingered robotic hands. The whole progress of solving dexterous manipulation with reinforcement learning is shown in Figure 4. In this section, we will discuss dexterous manipulation with reinforcement learning and its variations.

### Reinforcement Learning

The reinforcement learning problem is a kind of machine learning algorithm which learns mapping environment state to action and obtaining the maximum cumulative reward in the process of interaction with the environment. Q-learning is a traditional solution to the problem, however, it is not sufficient for more complicated problems today due to the high cost of solving the q-valued function with lots of states and actions. The combination of deep learning and reinforcement learning called deep reinforcement learning (DRL) was proposed for more complicated problems and it dominates the area now.

The method can be divided into the model-based method and model-free method, the difference between the two is whether a predictive model is used. The earliest model-based algorithm is Dyna (Sutton, 1990), where the model is learned by data from the real world and both the data from the real world and the learned model are used in the training process. There are some other model-based algorithms such as PILCO (Deisenroth and Rasmussen, 2011), M-PGPE (Mori et al., 2013), PEGASUS (Ng and Jordan, 2013), GPS (Levine and Abbeel, 2014), VPN (Oh et al., 2017), MVE (Feinberg et al., 2018), STEVE (Buckman et al., 2019), and MBPO (Janner et al., 2019). On the contrary, in the model-free method, the agent learns the strategy directly by interacting with the environment. The comparison between the model-based method and model-free method can be seen in Table 2. According to the characteristic of the model-based method and model-free method, the selection between the model-based method and model-free method is a crucial problem and should be taken into account.

**Table 2:** Classification and corresponding advantages and disadvantages of the RL methods.

**Classification**	**Advantages**	**Disadvantages**
Value-based RL	1. Easy to implement 2. High sample utilization	1. Poor performance in tasks of discontinuous and large state space 2. High bias
Policy-based RL	1. Easier to converge 2. More directly	1. Easy to converge to local optimum 2. High variance
Model-based RL	1. More data efficient 2. Faster convergence	1. Model accuracy has a big impact on learning tasks
Model-free RL	1. Easier to implement 2. No need of prior knowledge	1. Demanding much data 3. High risk of damage

Reinforcement learning also can be divided into three types according to the variables iterated in the learning process: value-based method, policy-based method, and actor-critic method. In the value-based method, the value function is learned and the policy is determined by a greedy strategy or a strategy. Deep Q-learning (DQN) (Mnih et al., 2015) and its variations (van Hasselt et al., 2015; Schaul et al., 2016; Wang et al., 2016) are typical model-free value-based method. Although DQN and its variants have achieved excellent performance in discrete action space problems such as video games, and even defeated human players by overwhelming advantage in some games, they cannot cope with the continuous action space problems that exist in many actual production and life such as dexterous manipulation.

Different from the value-based approach, the policy is straightly optimized in policy-based algorithms. REINFORCE (Williams, 1992) is a monumental algorithm which provides the state transition model-independent algorithm theoretically and becomes the starting point of many algorithm improvements. It plays a pioneering role in the algorithm system of policy gradient series represented by TRPO (Schulman et al., 2015) and PPO (Schulman et al., 2017). However, although TRPO and PPO algorithms have excellent hyperparameter performance and have gained attention in academic research as typical on-policy algorithms, many samples under the current policy need to be sampled for training and to ensure algorithm convergence each time the policy is updated. Therefore, the algorithms have low sampling efficiency and need a large amount of computational force to support, which greatly limits the popularization of the algorithms in the application field. A survey of the classification and corresponding comparison between the advantages and disadvantages of RL methods is shown in Table 2. Furthermore, a more detailed comparison between typical value-based algorithms and policy-based algorithms can be seen in Table 3.

**Table 3:** Comparison between typical value-based algorithms and policy-based algorithms.

**Algorithm**	**Main characteristic**	**Value-based/ policy-based**	**Limitations**
DQN (Mnih et al., 2015)	Approximating the optimal Q-value function with a deep convolutional neural network. Target-network and Experience replay	Value-based	Only capable of handling discrete and low-dimensional action spaces
Double DQN (van Hasselt et al., 2015)	Two networks are used for dealing with the overestimation problem of DQN	Value-based	
DQN with prioritized experience replay (Schaul et al., 2016)	Experience replay with priority is used to increase the learning utilization rate of samples and increase exploration	Value-based	
Dueling DQN (Wang et al., 2016)	V(s)+A(s, a) is used to replace Q(s, a) to alleviate the overestimation problem of DQN	Value-based	
REINFORCE (Williams, 1992)	The starting point of policy gradient algorithms	Policy-based	Low efficiency and high variance
TRPO (Schulman et al., 2015)	Finding the right step size to stably improve the policy	Policy-based	
PPO (Schulman et al., 2017)	An advanced version of TRPO which is easier to implement	Policy-based	

As we listed in Table 2, an important problem of the policy-based method is high variance and the combination of the value-based method and a policy-based method called the actor-critic method can solve this problem to some extent. The state-of-the-art algorithms at present are all under the actor-critic framework. The typical RL algorithms under the actor-critic framework are summarized in Table 4.

**Table 4:** Summary of typical algorithms under the actor-critic framework.

**Method**	**Main characteristic**	**Off-policy/** **On-policy**
A3C (Mnih et al., 2016)	Adopting asynchronous training framework	On-policy
DDPG (Lillicrap et al., 2015)	Able to deal with continuous space of action issues	Off-policy
TD3 (Fujimoto et al., 2018)	An advanced version of DDPG solving the problem of overestimation in actor-critic and addressing variance	Off-policy
SAC (Haarnoja et al., 2018)	Adopting Maximum Entropy Model to improve the robustness of the algorithm and speed up training	Off-policy

The actor-critic algorithm is mostly off-policy and can solve the problem of sampling efficiency through experience replay. However, the coupling of the policy update and value evaluation results in the lack of stability of the algorithm, especially the sensitivity to hyperparameters. In the actor-critic algorithm, it is very difficult to adjust parameters, and the algorithm is also difficult to reproduce. When it is promoted to the application field, the robustness of the algorithm is also one of the most concerning core issues. Commonly, the data of reinforcement learning are often incomplete, so we refer the readers to the following literature (Shang et al., 2019; Luo et al., 2020; Wu D. et al., 2020; Wu et al., 2020; Liu et al., 2021) for more details.

### Dexterous Manipulation With Multi-Fingered Robotic Hands Using RL From Scratch

**Figure 5:**
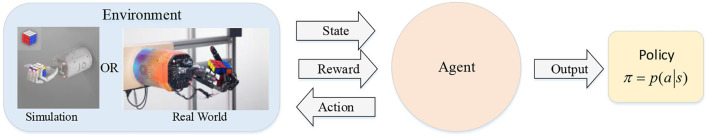
Basic process of learning dexterous manipulation by RL from scratch (part of this picture comes from Open et al., 2019).

The success in various complex tasks such as reorienting an object (Open et al., 2019), tool use (Rajeswaran et al., 2018), and playing the piano (Xu et al., 2021) has shown the power of reinforcement learning for dexterous manipulation. For dealing with the dexterous manipulation problem under the framework of RL, the problem is usually modeled as a Markov decision process (MDP), where the states can be the combination of internal states and external states, and the action is typically the motor commands. In a simulation, the states are available, however the needed elements for states cannot be obtained directly. Under that condition, visual sensors and tactile sensors are usually used for inferring the state or using the raw sensor data as the state (Katyal et al., 2016). The easiest way to think of is to train the agent from scratch. The basic process of learning dexterous manipulation by RL from scratch is depicted in Figure 5.

Although learning-based methods are appealing to roboticists for dealing with the dexterous manipulation problem, the need for large amounts of data has always been a major obstacle to the development of robotics. Hence, most researchers focused on enhancing the sample efficiency but from various angles. Some of the researchers focus on the algorithm itself and test only in simulation (Popov et al., 2017; Haarnoja et al., 2019; Omer et al., 2021). Popov et al. (2017) decouples the update from the frequency of interaction and trades off between the exploration and the exploitation by defining certain starting states and shaping reward effortfully. Haarnoja et al. (2019) improved the SAC for accelerating training and improving stability. Omer et al. (2021) present MPC-SAC combining the Model-Predictive Control (MPC) which is an offline learning method with online planning, which can be seen as a model-based RL method. Similarly, model-based methods are also adopted in (Kumar et al., 2016; Nagabandi et al., 2020). Different approximators such as time-varying linear-Gaussian (Kumar et al., 2016) and deep neural network (Nagabandi et al., 2020) are used, respectively. Moreover, the combination of local trajectory optimization and RL is also attractive (Lowrey et al., 2019; Charlesworth and Montana, 2021). Fakoor et al. (2020) centered around the instability problem in RL and reduced the complexity in the famous state-of-the-art RL algorithms. Some researchers also pay attention to the problem of sparse reward which is a common hindrance in RL causing sample inefficiency. To this end, HER is a widely used algorithm which learns from failures and can be combined with any RL algorithm. Li S. et al. (2019) just incorporate HER in the hierarchical RL framework to achieve the complex Rubik's cube task. The introduction of HER in RL can also be seen in (He et al., 2020; Huang et al., 2021).

Besides the problem of sample inefficiency, generalization is another major obstacle yet to be bordered. As a rule, multi-task RL is a popular concept to the researchers in autonomous robots (Hausman et al., 2018; He and Ciocarlie, 2021; Huang et al., 2021). Considering the inefficient exploration caused by the high DoF of the dexterous robotic hand, which means the high dimension of action space, He and Ciocarlie (2021) proposed a lower-dimensional synergy space and multi-task policy. In contrast to exploring in the raw action space with high dimension, exploring in the synergy space can improve the efficiency in exploring new environments or learning new tasks. Similarly, Hausman et al. (2018) presented embedding space to the same end. What is different is that Huang et al. (2021) focused on one task on various objects other than different tasks. With the help of a well-designed object representation and multi-task framework, the manipulation of 70 different objects can be realized by one policy model achieving similar or better results than single-task oracles. The success of this work is a big step toward making robotic hands intelligent.

The previously mentioned works were only tested in a simulation where data were easy to get, however, a great performance in simulation cannot guarantee the performance. Furthermore, the elements required for representing the state are not available in the real world, so sensors are necessary for representing the state. As a rule, the visual sensor is the main consideration. For instance, Haarnoja et al. (2019) adopted the raw image as a representation of the state. Experiences implicate that the introduction of tactile information can effectively improve the sample efficiency for training and the performance in dexterous manipulation tasks (Melnik et al., 2019). van Hoof et al. (2015) used the tactile sensor data and introduced the non-parametric relative entropy policy (NPREPS), which is well-suited to the sensor data. Falco et al. (2018) used the visual sensor and tactile sensor together. The visual sensor is used for representing the state in the RL process and the tactile sensor acts as feedback in a low-level reactive control aiming at avoiding slipping. Also, training on a real robotic hand usually costs time and requires human intervention. To alleviate the problem, Gupta et al. (2021) proposed a reset-free reinforcement learning algorithm. They pointed out that the learning of multi-task and sequencing them appropriately can solve the problem naturally. The algorithm achieved great performance both in simulation and the real world.

All the details of the above works are listed in Table 5, including the specific method, the environment (e.g., simulation or real world or from simulation to real world), the manipulator, sensors utilized, and the tasks.

**Table 5:** Overview of the dexterous manipulation solved by RL from scratch.

**References**	**Method**	**Manipulator**	**Sensors**	**Environment**	**Tasks**
Popov et al. (2017)	Improved DDPG	Jaco arm	-	Simulation only	Lego assembly
Fakoor et al. (2020)	DDPG++	ADROIT hand	-	Simulation only	Door opening
He and Ciocarlie (2021)	DisoSyn (based on PPO)	Shadow hand	-	Simulation only	Multi-tasks
Huang et al. (2021)	DDPG+HER+Multi-task learning	Shadow hand	-	Simulation only	In-hand rotation
Katyal et al. (2016)	DQN	Modular Prosthetic Limb (MPL)	-	Simulation only	In-hand manipulation
Li S. et al. (2019)	DDPG+HER	Shadow hand	-	Simulation only	Solving a 2*2*2 Rubik's Cube
Omer et al. (2021)	MPC-SAC	Dclaw and Shadow hand	-	Simulation only	Valve-turning and manipulating a cube
He et al., 2020	Soft HER	Shadow hand	-	Simulation only	Hand manipulate block and others
Xu et al. (2021)	SAC	Allegro hand	tactile sensors	Simulation only	Playing piano
Kumar et al. (2016)	RL with linear-Gaussian controllers (model-based RL)	Adroit platform	pressure sensors and piston length sensors	Simulation and real robot	Hand positioning and object manipulation
van Hoof et al. (2015)	NPREPS (van Hoof et al., 2015)	An under-actuated compliant robot hand	Tactile sensor	Real world	Rolling an object between fingertips
Nagabandi et al. (2020)	PDDM (model-based RL)	Shadow hand	Camera tracker	Real world	Baoding balls
Haarnoja et al. (2019)	SAC	Dclaw	Visual sensor	Real world	Valve rotation
Zhu H. et al. (2018)	TNPG	Dclaw and Allegro Hand	-	Real world	Valve Rotation and Door opening
Gupta et al. (2021)	MTRF	D'Hand	-	Real world	Pipe insertion and In-hand manipulation

### Dexterous Manipulation With Multi-Fingered Robotic Hands Using Reinforcement Learning From Demonstration

Apart from improvement on the RL algorithm, some researchers were inspired by the way learners paid attention to learning from demonstrations, which is also called imitation learning. An intuitive idea is following the expert demonstrations in a supervised way, namely behavior cloning. However, the policy depends on the expert data too much in this way. Another common method in imitation learning is inverse reinforcement learning where the reward function is learned. The introduction of demonstration data in reinforcement learning is an effective approach for enhancing the sample efficiency and the generalization performance in behavior cloning only.

**Figure 6:**
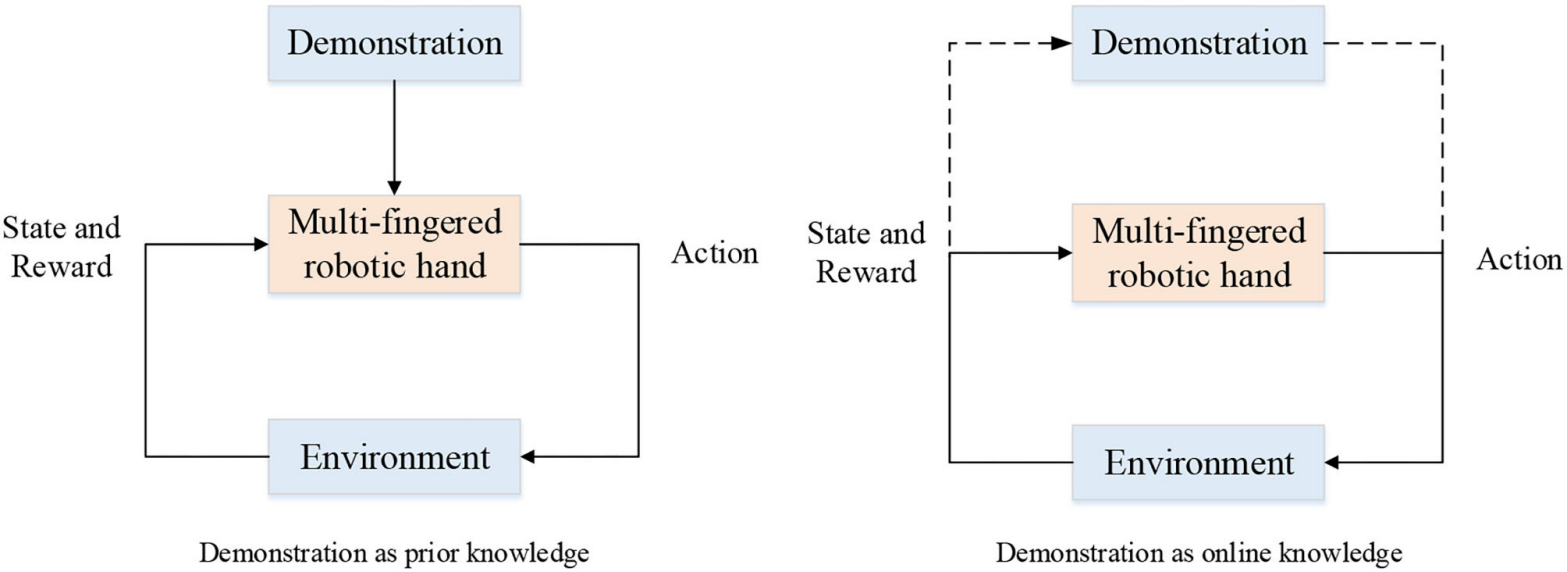
Two types of combination of RL and demonstration.

The sources of demonstrations can be kinesthetic teaching, teleoperation (Zahlner S. et al., (n.d.); Handa et al., 2019; Li T. et al., 2019; Li et al., 2020), raw video, and so on. The problem of learning from demonstration has been studied a lot in recent years and a comprehensive survey can be seen in Ramírez et al. (2021). Ramírez et al. (2021) divided the use of the demonstrations into two types of knowledge: prior knowledge and online knowledge. In the case of the former, the demonstration data were stored before the RL process and acted as source of knowledge such as being added to the reward function for bringing the policy closer to the demonstration. In the case of the latter, the demonstrations are used occasionally to provide a trajectory. The process of the two types of combination can are depicted in Figure 6.

Here we follow the same sort of classification and go further into the application in dexterous manipulation with multi-fingered robotic hands. In the first class, the demonstrations can be utilized in various ways. For instance, a kinesthetic demonstration is adopted as the desired position trajectory as prior knowledge to get an initial force profile and then optimized through RL (Kalakrishnan et al., 2011). Prieur et al. (2012) decomposed the whole dexterous manipulation problem into a sequence of canonical-grasp-type identified in the humans. Although the introduction of human motion helps the problem, the motion of the robot is limited to these grasp types. Conversely, an “object-centric” demonstration which only demonstrated the motion of the object was adopted due to the special end-effector used in the work of Gupta et al. (2016). Also, the demonstrations can be used to per-train an initial policy (Rajeswaran et al., 2018; Alakuijala et al., 2021). For further improving the sample efficiency, Alakuijala et al. (2021) adopted residual reinforcement learning.

The demonstration data also can be stored to provide an auxiliary part in the reward function. Considering the state-action pairs trajectories are not available all the time, Radosavovic et al. (2020) proposed State-Only Imitation Learning (SOIL) where an inverse model is also learned to infer the action for the demonstrated state. An important work combining reinforcement learning and imitation learning is generative adversarial imitation learning (GAIL), which is used widely in the domain of dexterous manipulation (Zhu Y. et al., 2018) DexMV (Qin et al., 2021). Orbik et al. (2021) adopted the inverse reinforcement learning method and improved the original algorithm to the problem that the learned rewards are strongly inclined to the demonstrated actions using statistical tools for random sample generation and reward normalization.

In the second class, the demonstrations are usually stored in the replay buffer and act as online knowledge to provide guidance. Jeong et al. (2021) used a set of waypoints (pose) tracking controllers as a suboptimal expert. The demonstration data were used in the exploration process occasionally by intertwining with the online interaction data. And, the combination of the exploration strategy and the Relative Entropy Q-Learning (REQ) algorithm called REQfSE outperformed the DDPG from demonstrations (DDPGfD) (Vecerik et al., 2018) and MPOfD (Jeong et al., 2019) on several tasks, such as single-arm stacking in the simulation environment. Garcia-Hernando et al. (2020) used the imperfect estimated hand pose as a demonstration. The action was combined between the hand pose estimation from inverse kinematics (IK) and the output of the residual policy network for imitating the hand pose in the real world more accurately. Because of its sheer volume and availability, a raw video is an appealing form of demonstration data. DexMV (Qin et al., 2021) just adopted this idea. They estimated the hand-object pose from raw video and used the estimation as demonstration data to learn robust policy with imitation learning. This work is a great beginning for further research in dexterous manipulation or any other vision-based research related to imitation learning.

According to the analysis previously, a summary of the works in this section is listed in Table 6.

**Table 6:** Overview of the dexterous manipulation solved by RL with a demonstration.

**References**	**Method**	**Manipulator**	**Sensors**	**Environment**	**Form of demonstration**	**Tasks**
Qin et al. (2021)	DexMV	Adroit Hand	-	Simulation only	Raw video	Relocating, pouring and placing inside
Zhu H. et al., 2018	DAPG	Dclaw and Allegro Hand	-	Real robot	kinesthetic teaching	Valve Rotation, Valve Rotation and Door opening
Orbik et al. (2021)	IRL	Adroit Hand	-	Simulation only	CyberGlove	Object relocation, tool use, in-hand manipulation and door opening
Rajeswaran et al. (2018)	DAPG	Adroit hand	-	Simulation only	CyberGlove	Object relocation, tool use, in-hand manipulation and door opening
Gupta et al. (2016)	Learning from demonstrations algorithm based on the GPS	RBO Hand 2	Phase space Impulse system	Real robot	LED marker tracking the motion of the object demonstrated by human	Turning a valve, pushing beads on an abacus, and grasping a bottle from a table
Jeong et al. (2021)	REQfSE	Bimanual Shadow Hand	-	Simulation only	Waypoint controllers	LEGO stacking
Alakuijala et al. (2021)	RRLfD	Adroit Hand	-	Simulation only	Script or a previously trained RL agent	Object relocation, tool use, in-hand manipulation and door opening
Radosavovic et al. (2020)	SOIL	Adroit Hand	-	Simulation only	virtual reality headset and a motion capture glove	Object relocation, tool use, in-hand manipulation and door opening

### Dexterous Manipulation From Simulation to Real Robotics

**Figure 7:**
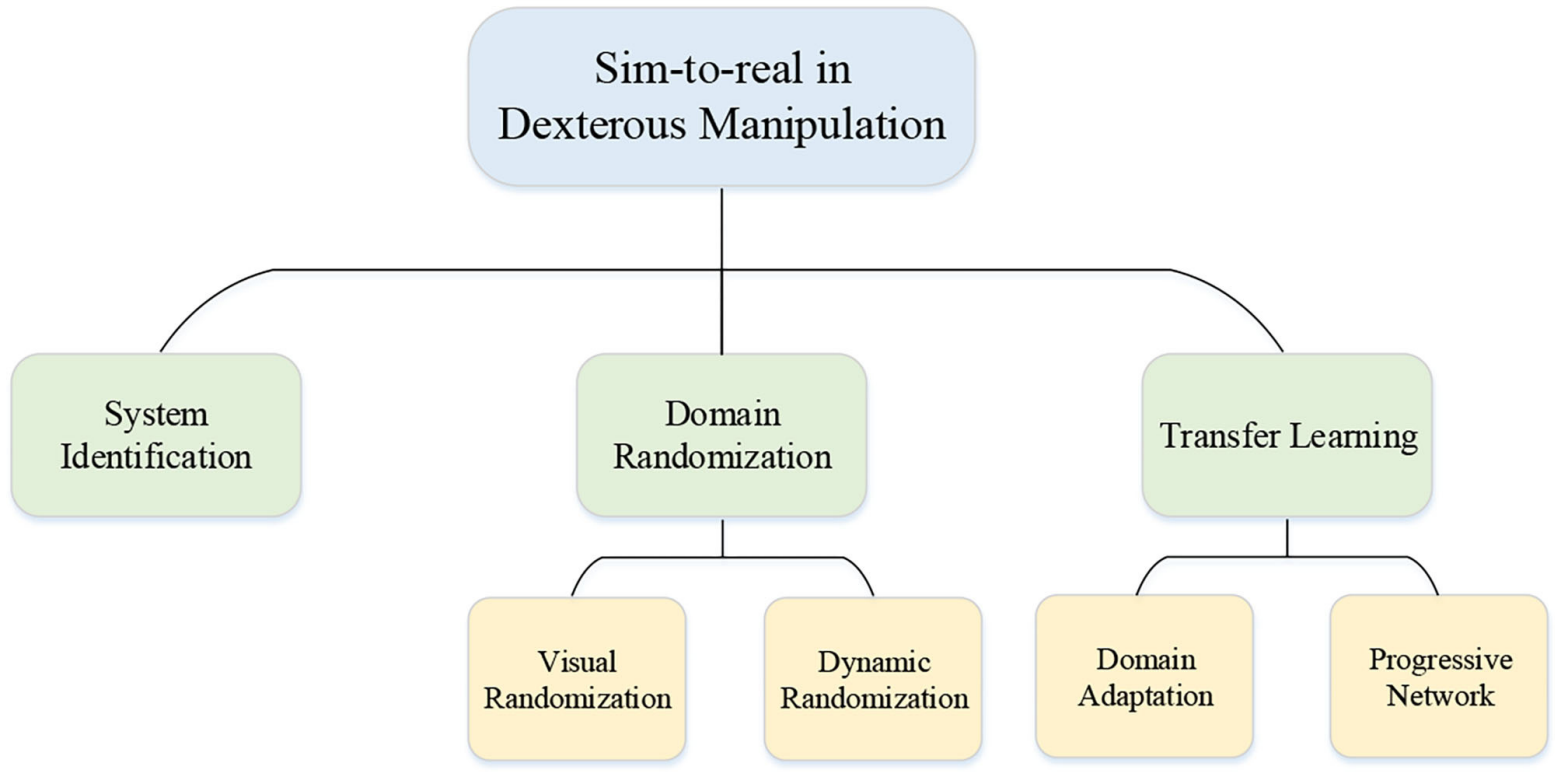
Category of approaches for sim-to-real in this domain.

Benefiting from the parallel and powerful computations, collecting data in simulators is easier and safer than that in the real world. Therefore, learning in simulation and then transferring the learned policy to a real robot is appealing to researchers. However, the discrepancies between simulation and real robot make the transformation challenging, which are generally called “reality gaps” including dynamics differences of engines, and so on. Transforming the policy directly to the real world may cause various consequences, the lesser of which is a decline in success and the more serious of which is the instability of the system that may destroy the robotic hands or the environment. Hence, closing the reality gap is the main issue when mentioning the sim-to-real problem. For narrowing the gap, some researchers focused on building higher fidelity simulators such as MuJoCo (Todorov et al., 2012), PyBullet (Coumans and Bai, 2016), and Gazebo (Koenig and Howard, 2004). However, it is generally accepted that the improvement of simulators will not bridge the gap completely. The typical approaches for bridging the reality gap in the domain of dexterous manipulation with multi-fingered robotic hands with RL are depicted in Figure 7 and the application of these approaches in this domain will be introduced in the following part.

The sim-to-real problem is not unique to the field of reinforcement learning or dexterous manipulation, but general problem in machine learning. The main approaches widely used for closing the reality gap are system identity, domain randomization, and transfer learning including domain adaptation and progressive networks. However, on account of that the models of multi-fingered robotic hands and the complex environment are impossible to be accurately built in the simulators. The simplest system identity method is not desirable and other approaches must be considered. Instead of building an accurate model of the real world in system identity, the main idea of domain randomization is to randomize the simulation with disturbance. The elements can be randomized and include many aspects which can be roughly divided into parts visual randomization and dynamic randomization. For instance, the randomization of lighting, textures of the object, and the positions of the cameras belong to visual randomization, and the randomization of surface friction coefficients, the contact model, and the object mass belong to dynamics randomization. Through exposure to various environments, the learner trained in simulation can adapt to a wide range of environments. So for the learner, the real world is just a disturbed environment. More details of the sim-to-real problem can be seen in Zhu et al. (2021).

The idea of randomization is widely adopted in the sim-to-real problem of dexterous manipulation (Allshire et al., 2021). For instance, in the work of Zhu H. et al. (2018), only visual randomizations were adopted for zero-shot transfer from simulation to reality. Unlike learning policies robust to senses with high variation mentioned before, Kumar et al. (2019) focused on the variation of object appearance and geometry such as object mass, friction coefficients between the fingers and object, PD gains of the robot, and damping coefficients of the robot joints. Visual sensing is used to abstract away the uncertainties into a succinct set of geometric features and tactile sensors are adopted to compensate for the inaccurate approximation. After training in the simulation, a zero-shot transfer is achieved on the real robot for a grasping task. Similarly, the idea of randomization of friction, object mass, and object scale was also adopted by Allshire et al. (2021), where the training process was carried out in IsaacGym (Liang et al., 2018). The notable work accomplished by OpenAI (Andrychowicz et al., 2020) also adopted the approach of domain randomization to transfer the policy learned in the MuJoCo simulator to a real Shadow hand. Apart from visual randomizations and physics randomizations, a lot of other randomizations were adopted. Through extensive randomizations, the learned policy got a great performance in the real robot system without any fine-tuning. The success of this work demonstrates that the gap between the simulation and reality can be narrowed to a usable level. Later, they improved the algorithm to solve a more complicated task of solving a Rubik's cube (Open et al., 2019). The concept of domain randomization was also considered, however, they improved it for a better format, namely automatic domain randomization (ADR). The main improvement compared to classic domain randomization lies in the automatic change of the distribution ranges leaving out tedious manual tunning. Furthermore, unlike fixed distribution ranges in classic domain randomization, the distribution ranges are allowed to change in ADR instead.

The intuition of transfer learning is leveraging the data from a source domain where the data are abundant and sufficient to help learn a robust policy in the target domain with little data. The progressive network proposed by Deepmind (Rusu et al., 2016) is a unique structure of a neural network with the ability to use the knowledge of the previous task for the new task without catastrophic forgetting. Later, they adopted this idea for robot manipulation (Rusu et al., 2017). Also, some researchers focused on the RL algorithm itself such as hierarchical decomposition RL (Fernandes Veiga et al., 2020) to bridge the reality gap. Considering that the privileged state information is not available in reality, researchers usually used rendered pictures as observation (Open et al., 2019; Andrychowicz et al., 2020). However, the accurate privileged state information in a simulator can accelerate the training process and get a better policy. An idea of teacher-student training which transfers the better teacher policy to a student policy that only uses sensory inputs was adopted in (Chen et al., 2021) for accelerating the training process in real world. A summary of the works in this section is listed in Table 7.

**Table 7:** Overview of the dexterous manipulation from simulation to reality.

**References**	**RL**	**Sim2Real**	**Manipulator**	**Simulator**	**Sensor**	**Task**
Andrychowicz et al. (2020)	PPO	Domain randomization	Shadow hand	MuJoCo physics engine (Todorov et al., 2012)	3D tracking system and RGB cameras	Manipulating a block
Open et al. (2019)	PPO	Automatic domain randomization	Shadow hand	MuJoCo physics engine (Todorov et al., 2012)	3D tracking system and RGB cameras	Solving a Rubik's cube
Zhu Y. et al. (2018)	A method based on GAIL(IL)+PPO(RL)	Domain randomization	Jaco arm	MuJoCo physics engine (Todorov et al., 2012)	RGB cameras	Block lifting and stacking
Kumar et al. (2019)	Contextual RL (PPO)	Domain randomization	Allegro hand	-	RGB cameras and Tactile sensor	Grasping
Allshire et al. (2021)	PPO	Domain randomization	TriFinger	IsaacGym	RGB cameras	In-hand manipulation
Rusu et al. (2017)	A3C	Progressive net	Jaco arm	MuJoCo physics engine (Todorov et al., 2012)	RGB cameras	Reaching to a visual target
Fernandes Veiga et al. (2020)	Hierarchical control (RL+ tactile feedback control)	Hierarchical RL	Allegro hands	PyBullet Coumans and Bai (2016) simulation environment	Tactile sensor	In-hand manipulation

## Challenges and Future Research Directions

Although the methods mentioned in this paper already solved part of the dexterous manipulation problems, we are still a long way from making the robotic hands as dexterous as human hands. And the complexity of the multi-fingered robotic hand system, such as uncertain models, dimensional disaster has restricted the development of RL in dexterous manipulation with multi-fingered robotic hand domain. In general, the challenges the community is facing in this domain are as follows:

- *Sample inefficiency:* The demand of more data limits the tasks which can be solved by RL from scratch to a narrow scope.
- *Tradeoff between exploration and exploitation:* Through exploration, the robot can get more information about the environment, but random behavior may not get rewards in tasks with sparse rewards, which would not make the algorithm converge. On the other hand, exploitation gives more knowledge about the environment to make the best decision, but the deficiency of information may lead to a locally optimal solution. Therefore, two questions should be answered: how to explore efficiently and effectively and when to transition from exploration to exploitation.
- *Choosing of suitable manipulator:* High stiffness improves precision but lacks flexibility and may damage the environment, whereas low stiffness (i.e., soft robotic fingers) improves robustness but suffers from inaccuracy. Furthermore, there is a tradeoff between dexterity and control simplicity.
- *Reality gap:* Despite the methods such as domain randomization mitigating the gap to the extent of one-shot transferring, the reality gap is also a problem that cannot be ignored.
- *High cost of time and resources:* A long time is required for obtaining a robust policy in terms of large-scale experiments. Furthermore, the multi-fingered robotic hands are so expensive and fragile that maintaining and repairing these robotic hands costs much. The immense requirement keeps such success at the laboratory level.
- *Poor generalization ability:* In general, the learned policy only fits to the specific task and robot, generalizing the policy to different robotic hands and tasks remains challenging.
- *Hardware limitations:* The high demands on the sensors makes it challenging to achieve the dexterity of a human hand. Moreover, the rigid plastic and metal components of the current robotic hands are the main reasons for the lack of dexterity. Although a variety of commercial products are available, their touch sensors are rigid and their placement is limited to the fingertips and along the limb segments, which is not desirable.
- *Complex manipulation is still unavailable:* Although some simple tasks such as pick-and-place, throwing, sliding, pivoting, and pushing can be done, some more complex tasks, especially those that change the shape of objects (cutting, crushing) are unavailable. A model of the deformation and advanced perception to monitor the changes is required.

To meet the challenges and accelerate the process of robotic hands intelligence, the future directions for researchers can be summarized below:

♦ *More advanced simulators:* Although some great simulators can be as fast as realistic or even faster in many cases, the existing simulators have certain limitations for emulating some elements of the environment. The more advanced the simulators are, the better performance in transferring the policy learned in simulation to reality. Furthermore, more manipulation scenarios are more desirable.
♦ *Fusion of sensors:* For more accurate information about the system, the visual sensing information used widely in the previous works is not sufficient, so multimodal sensory signals which include, but are not limited to, tactile and temperature signals should be used to represent the state of the system.
♦ *Improvement of the algorithm:* The rewards in the existing algorithms are typically designed carefully and only simple tasks such as reaching and pushing can be accomplished with sparse rewards. For this problem, informed exploration may be helpful. Furthermore, the adaptation to the variations of both the robot variations and variations in the environment is essential for working gracefully. Therefore, more sophisticated methodologies must be found for dealing with these problems and accelerating the training process.
♦ *Semantic understanding:* Learning to understand the environment and the task and following the human order are also vital skills for a robot to work with more intelligence. For a given order from voice or other forms, a robot should know what to do and how to do the task.
♦ *Improvement of robotic hands:* Although there have been many robotic hands in this domain, the limited dexterity of the simple end-effector and the fragility and characteristics that are not conducive to controlling the complex dexterous multi-fingered hands hinder the development of the domain. The tradeoff of the dexterity and the complexity of control should be balanced.
♦ *Manipulation in media such as water or oil:* The existing successful examples of dexterous manipulation are all in the air. However, for some special tasks, such as underwater operation, the ability to manipulate in the water is especially important.
♦ *Deeper study in basic theoretical:* Currently, the model of soft point-contact and stability rules for both point contacts and surface contacts, which are vital for modeling the system, are not available. Although the model is not essential for a learning-based approach, the emphasis on theory may be conducive for a better simulator.

## Conclusion

In this paper, we present a brief overview of the reinforcement learning solutions for dexterous manipulation, focusing mainly on reinforcement learning, reinforcement learning from demonstration, and transfer learning from simulation to reality. The application of reinforcement learning in dexterous manipulation with the multi-fingered robotic hand is mostly hampered by the high cost of collecting sufficient data for a great policy. At present, the common and effective ways for mitigating data inefficiency issues are learning from demonstration and transferring the learned policy in simulation to the real world. However, compared with the tasks that humans can handle easily, what the multi-fingered robotic hands can do is still very limited. Despite this, we believe that the reinforcement learning-based solution can do a lot as the research goes further.

## Author Contributions

PW contributed to the conception of the study and revised the manuscript critically for important intellectual content. CY collected related literature and wrote the manuscript. Both authors reviewed the results and approved the final version of the manuscript.

## Funding

This work was supported in part by the National Natural Science Foundation of China under Grants (91748131, 62006229, and 61771471), in part by the Strategic Priority Research Program of Chinese Academy of Science under Grant (XDB32050106), and in part by the InnoHK Project.

## Conflict of Interest

The authors declare that the research was conducted in the absence of any commercial or financial relationships that could be construed as a potential conflict of interest.

## Publisher's Note

All claims expressed in this article are solely those of the authors and do not necessarily represent those of their affiliated organizations, or those of the publisher, the editors and the reviewers. Any product that may be evaluated in this article, or claim that may be made by its manufacturer, is not guaranteed or endorsed by the publisher.
